# Energy stress-induced PKCζ S-glutathionylation is essential for LKB1 cytoplasmic translocation and AMPK activation

**DOI:** 10.1093/lifemeta/loaf027

**Published:** 2025-07-16

**Authors:** Junjie Fei, Shuhan Yu, Mingzhe Xu, Yang Liu, Guoqiang Wang, Xueqing Li, Xinyue Yu, Yifan Zhang, Wenhua Zhang, Yang Wang, Mengmeng Niu, Yujun Zhang, Yang Cao, Zhi-Xiong Jim Xiao, Yong Yi

**Affiliations:** Center of Growth, Metabolism and Aging, Key Laboratory of Bio-Resource and Eco-Environment of Ministry of Education, College of Life Sciences, Sichuan University, Chengdu, Sichuan 610064, China; Center of Growth, Metabolism and Aging, Key Laboratory of Bio-Resource and Eco-Environment of Ministry of Education, College of Life Sciences, Sichuan University, Chengdu, Sichuan 610064, China; Center of Growth, Metabolism and Aging, Key Laboratory of Bio-Resource and Eco-Environment of Ministry of Education, College of Life Sciences, Sichuan University, Chengdu, Sichuan 610064, China; Center of Growth, Metabolism and Aging, Key Laboratory of Bio-Resource and Eco-Environment of Ministry of Education, College of Life Sciences, Sichuan University, Chengdu, Sichuan 610064, China; Center of Growth, Metabolism and Aging, Key Laboratory of Bio-Resource and Eco-Environment of Ministry of Education, College of Life Sciences, Sichuan University, Chengdu, Sichuan 610064, China; Center of Growth, Metabolism and Aging, Key Laboratory of Bio-Resource and Eco-Environment of Ministry of Education, College of Life Sciences, Sichuan University, Chengdu, Sichuan 610064, China; Center of Growth, Metabolism and Aging, Key Laboratory of Bio-Resource and Eco-Environment of Ministry of Education, College of Life Sciences, Sichuan University, Chengdu, Sichuan 610064, China; Center of Growth, Metabolism and Aging, Key Laboratory of Bio-Resource and Eco-Environment of Ministry of Education, College of Life Sciences, Sichuan University, Chengdu, Sichuan 610064, China; Center of Growth, Metabolism and Aging, Key Laboratory of Bio-Resource and Eco-Environment of Ministry of Education, College of Life Sciences, Sichuan University, Chengdu, Sichuan 610064, China; Center of Growth, Metabolism and Aging, Key Laboratory of Bio-Resource and Eco-Environment of Ministry of Education, College of Life Sciences, Sichuan University, Chengdu, Sichuan 610064, China; Center of Growth, Metabolism and Aging, Key Laboratory of Bio-Resource and Eco-Environment of Ministry of Education, College of Life Sciences, Sichuan University, Chengdu, Sichuan 610064, China; Center of Growth, Metabolism and Aging, Key Laboratory of Bio-Resource and Eco-Environment of Ministry of Education, College of Life Sciences, Sichuan University, Chengdu, Sichuan 610064, China; Center of Growth, Metabolism and Aging, Key Laboratory of Bio-Resource and Eco-Environment of Ministry of Education, College of Life Sciences, Sichuan University, Chengdu, Sichuan 610064, China; Center of Growth, Metabolism and Aging, Key Laboratory of Bio-Resource and Eco-Environment of Ministry of Education, College of Life Sciences, Sichuan University, Chengdu, Sichuan 610064, China; State Key Laboratory of Biotherapy, West China Hospital, Sichuan University, Chengdu, Sichuan 610041, China; Center of Growth, Metabolism and Aging, Key Laboratory of Bio-Resource and Eco-Environment of Ministry of Education, College of Life Sciences, Sichuan University, Chengdu, Sichuan 610064, China

**Keywords:** energy stress, ROS, PKCζ, S-glutathionylation, LKB1, AMPK

## Abstract

Energy stress triggers the activation of AMP-activated protein kinase (AMPK) via phosphorylation mediated by liver kinase B1 (LKB1). A pivotal step during this process is the translocation of protein kinase C zeta (PKCζ) to the nucleus, where it facilitates the phosphorylation and subsequent nuclear export of LKB1 to the cytosol. However, the mechanism(s) by which PKCζ translocates to the nucleus remains elusive. Here we demonstrate that energy stress, including glucose starvation or metformin treatment, elevates cellular reactive oxygen species (ROS) that promotes PKCζ nuclear import to promote LKB1 cytoplasmic translocation and subsequent AMPK activation both *in vitro* and *in vivo*. Mechanistically, we show that energy stress-induced ROS promotes the S-glutathionylation of PKCζ at Cys48, and enhances the interaction of PKCζ with karyopherin subunit alpha 2 (KPNA2), a key nuclear transport protein, thereby facilitating PKCζ nuclear translocation and the phosphorylation of LKB1 at Ser428, consequently leading to LKB1 cytoplasmic translocation and activation of AMPK. Importantly, the reduction of ROS significantly augments the high-fat diet-induced lipid accumulation in mouse liver and reduces the hypoglycemic efficacy of metformin in an AMPK-dependent manner. Together, these results establish a critical role of energy stress-induced PKCζ S-glutathionylation in LKB1 cytoplasmic translocation, highlighting the activation of the ROS−PKCζ−KPNA2−LKB1 axis as a vital mechanism for AMPK activation in response to energy stress.

## Introduction

AMP-activated protein kinase (AMPK) plays a critical role in maintaining cellular energy homeostasis via the regulation of a series of biological processes, including glucose metabolism, lipid biogenesis, and protein synthesis [1]. AMPK is a heterotrimer consisting of three subunits (α, β, and γ). The α subunit contains the catalytic kinase domain, and the β subunit serves as a scaffold protein important for heterotrimer formation. The γ regulatory subunit binds AMP, resulting in conformation changes of AMPK and exposing T172 for phosphorylation, a critical step for activation of AMPK kinase activity [2]. Metabolic stresses, such as glucose deprivation, hypoxia, and other means of accelerating ATP consumption, result in an increased pool of AMP, which in turn binds to AMPKγ, leading to the phosphorylation of AMPKα at Thr172 by the upstream kinase LKB1 [3, 4]. AMPK activation can be independent of AMP, as evidenced by the observation that the deprivation of fructose-1,6-diphosphate or inactivation of aldolase can promote AMPK−AXIN−LKB1 complex formation to active AMPK in an LKB1-dependent manner [5]. However, LKB1 is localized predominantly in the nucleus, whereas AMPK is localized mainly in the cytoplasm [6]. Therefore, nuclear LKB1 must be exported from the nucleus into the cytoplasm to phosphorylate and activate AMPK. It is reported that the phosphorylation of LKB1 at S428 is essential for LKB1 cytoplasmic translocation through the Mouse protein 25 (MO25)−LKB1−STE-20-related kinase adaptor protein (STRAD) complexes [6]. In addition, the orphan nuclear receptor Nur77 (nuclear receptor subfamily 4 group a member 1, NR4A1) can bind to and sequester LKB1 in the nucleus via inhibition of LKB1 phosphorylation at S428 [7].

Reactive oxygen species (ROS), including hydrogen peroxide (H_2_O_2_), superoxide (O^2−^), and hydroxyl (OH^−^), are important signaling molecules in the regulation of various biological processes [8]. It has been documented that energy stress promotes both AMPK activation and ROS accumulation [9], raising an interesting possibility that ROS may play a role in the activation of AMPK in response to energy stress. Accumulating evidence indicates that ROS can promote reversible and irreversible modifications of cysteine (thiol) residues of targeted proteins, including disulfide bond formation (S-S), glutathionylation (S-S-G), sulfenylation (SOH), sulfinic acid (SO_2_H), and sulfonic acid (SO_3_H) [10]. We have previously shown that ROS can trigger homodimerization of the ubiquitin-specific protease 5 (USP5) via Cys195 disulfide bond formation, thereby stabilizing USP5 and enhancing Kras-induced tumorigenicity [11]. ROS can promote S-glutathionylation of phosphatase and tensin homologue deleted fromchromosome 10 (PTEN) to inactivate PTEN upon ATP treatment [12]. Furthermore, ROS can induce rapid S-glutathionylation of glyceraldehyde-3-phosphate dehydrogenase (GAPDH) to promote its nuclear translocation [13]. In addition, ROS promotes S-glutathionylation of the fatty acid-binding protein 5 (FABP5) at Cys127 to facilitate its fatty acid binding ability and nuclear translocation [14]. These findings underscore the pivotal role of S-glutathionylation in modulating protein functionality and subcellular localization.

In this study, we show that energy stress elevates ROS, which in turn promotes S-glutathionylation of protein kinase C zeta (PKCζ) at Cys48, which facilitates the formation of the PKCζ−karyopherin subunit alpha 2 (KPNA2) protein complex and subsequent nuclear translocation of PKCζ. This process triggers the LKB1 phosphorylation at Ser428, leading to its translocation from the nucleus to the cytoplasm and consequently resulting in the activation of AMPK.

## Results

### Energy stress promotes LKB1 cytoplasmic translocation and AMPK activation through the elevation of ROS

Energy stress leads to the exhaustion of cellular ATP and an increase of AMP, which binds to the γ-subunit of the AMPK trimeric complex, resulting in the confirmation change and exposing Thr172 of AMPKα, an essential step for subsequent phosphorylation and activation by liver kinase B1 (LKB1) [1, 15, 16]. However, LKB1 is predominantly located in the nucleus [6]. Therefore, nuclear LKB1 must be exported into the cytosol to activate AMPK upon energy stress. However, the underlying mechanism(s) remains elusive.

**Figure 1:**
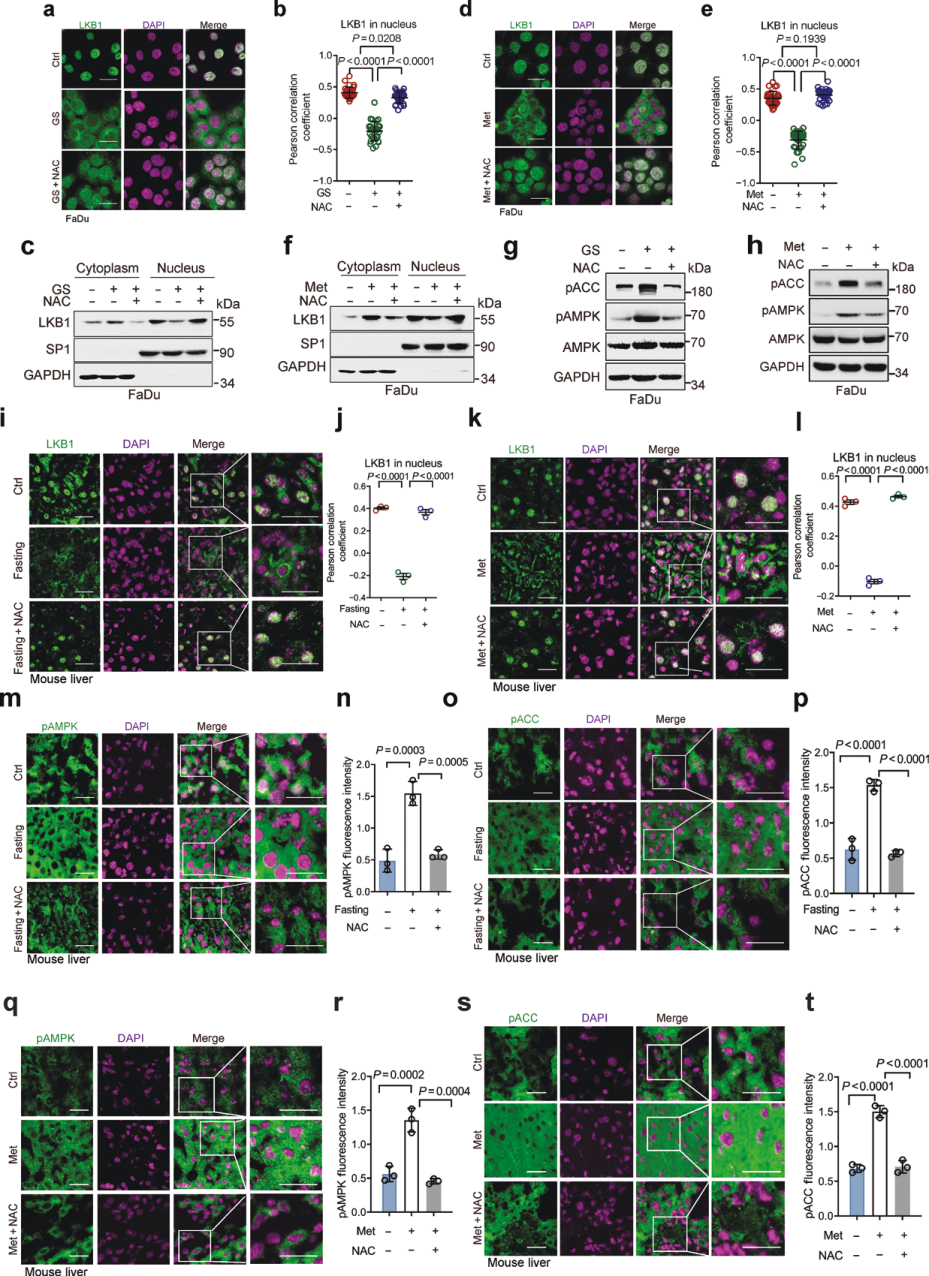
Energy stress-induced ROS is required for LKB1 cytoplasmic translocation and AMPK activation. (a−f) Immunofluorescence staining and cellular fractionation assays of cells treated with NAC or metformin (Met) to detect the cytoplasmic translocation of LKB1. FaDu cells were treated with or without NAC (10 mmol/L, and hereafter) for 10 h, followed by culture in 0 mg/mL glucose condition (glucose starvation, GS) in the presence or absence of NAC for an additional 2 h (a and c), or were treated with Met (10 mmol/L, and hereafter) in the presence or absence of NAC under 4.5 mg/mL glucose condition for 12 h (d and f). Cells were subjected to immunofluorescence staining analyses (a and d) or were subjected to cell fractionation and western blot analyses (c and f). The co-localization between LKB1 and DAPI (as analyzed by Pearson’s correlation coefficient) was quantified and statistically analyzed (b and e). A total of 30 cells derived from three independent experiments were randomly chosen and subjected to quantification analyses. (g) FaDu cells were treated with or without NAC for 10 h, followed by culture in GS condition in the presence or absence of NAC for an additional 2 h. Cells were subjected to western blot analyses. (h) FaDu cells were treated with or without Met in the presence or absence of NAC for 12 h. Cells were subjected to western blot analyses. (i and j) Immunofluorescence staining of liver sections from mice treated with NAC and following fasting. The 6-week-old male C57BL/6 mice (*n* = 3/group) were intraperitoneally (i.p.) injected with NAC (200 mg/kg) daily. On day 7 after injection, mice were fasted for 16 h before being sacrificed. Mouse liver sections were subjected to immunofluorescence staining (i). Pearson’s correlation coefficient was used to qualify the co-localization of LKB1 and DAPI, using images derived from immunostained liver samples (j). (k and l) Immunofluorescence staining of liver sections from mice treated with NAC and/or Met. The 6-week-old male C57BL/6 mice (*n* = 3/group) were i.p. injected with NAC (200 mg/kg) and/or Met (50 mg/kg) daily. On day 14 after injection, mice were sacrificed. Mouse liver sections were subjected to immunofluorescence staining (k). Pearson’s correlation coefficient was used to qualify the co-localization of LKB1 and DAPI, using images derived from immunostained liver samples (l). (m−p) Mouse liver sections derived from (i) were subjected to immunofluorescence staining (m and o). The pAMPK and pACC levels were quantified and statistically analyzed (n and p). (q−t) Mouse liver sections derived from (k) were subjected to immunofluorescence staining (q and s). The pAMPK and pACC levels were quantified and statistically analyzed (r and t). Data were presented as mean ± SD (b and e) or SEM (j, l, n, p, r, and t). Comparisons were performed with one-way ANOVA with Tukey’s test (b, e, j, l, n, p, r, and t). Scale bar, 25 μm. pAMPK: phospho-AMPK (Thr172); pACC: phospho-ACC (Ser79).

It is well-known that energy stress, such as glucose starvation or metformin treatment, is often associated with elevated levels of ROS [9, 17], as also evidenced in our experimental systems (Supplementary Fig. S1a and c). We thus investigated the role of ROS in energy stress-induced LKB1 cytoplasmic localization and AMPK activation. The results showed that glucose deprivation significantly promoted cytoplasmic translocation of LKB1 (Fig. 1a–c), consistent with a previous report [6]. However, the reduction of ROS by N-acetylcysteine (NAC), a ROS scavenger, effectively blocked glucose starvation-induced LKB1 cytoplasmic translocation, as evidenced by immunofluorescent and cellular fractionation assays (Fig. 1a–c; Supplementary Fig. S1a). Similar effects were observed from the treatment of metformin (Fig. 1d–f; Supplementary Fig. S1c). Furthermore, glucose starvation- or metformin-induced AMPK activation, as evidenced by the upregulation of phosphorylated Thr172 of AMPK (pAMPK) and phosphorylated Ser79 of acetyl-CoA carboxylase (ACC) (pACC) *in vitro,* could be markedly inhibited by NAC treatment (Fig. 1g and h; Supplementary Fig. S1b and d). Consistently, vitamin E and glutathione (GSH), two well-known ROS reducers, could also notably suppress glucose starvation- or metformin-induced AMPK activation (Supplementary Fig. S1e−h). Importantly, either fasting or metformin treatment of mice led to upregulated LKB1 cytoplasmic translocation and AMPK activation in mouse liver samples, which could be completely blocked by NAC treatment (Fig. 1i–t; Supplementary Fig. S1i−k). These results indicate that ROS is vital in energy stress-induced LKB1 cytoplasmic translocation.

Next, we investigated whether ROS-induced LKB1 cytoplasmic translocation is essential for AMPK activation upon energy stress. NAC had little effect on AMPK activation induced by either glucose starvation or metformin treatment in LKB1-null A549 cells (Supplementary Fig. S2a and b) , suggesting that the effect of ROS on AMPK activation is LKB1 dependent. Consistently, the reduction of ROS by NAC had little effect on CaCl_2_- or calcium ionophore A-23187-induced AMPK activation (Supplementary Fig. S2c and d), another important pathway of AMPK activation involved in a Ca^2+^/CaM-dependent kinase (CaMKK2) [18]. In addition, NAC did not affect the activity of a constitutively active AMPKα1 mutant (Aα1-CA), which is LKB1 independent due to the phosphorylation mimic T172D mutation [19] (Supplementary Fig. S2e). These results provide compelling evidence that LKB1 is essential for ROS-induced AMPK activation upon energy stress.

### Energy stress-induced ROS promotes PKCζ nuclear translocation to facilitate LKB1 phosphorylation and cytoplasmic translocation

**Figure 2:**
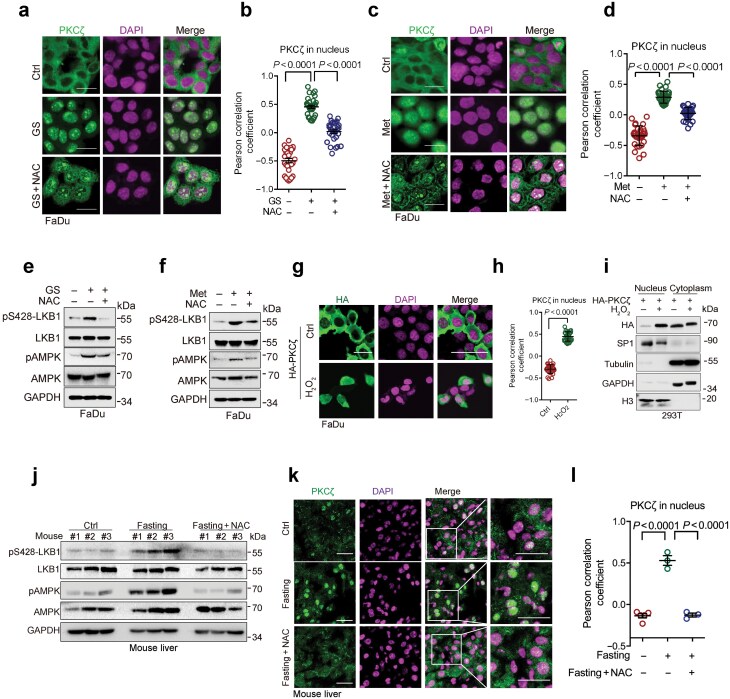
Energy stress-induced ROS promotes PKCζ nuclear translocation to facilitate LKB1 phosphorylation at Ser428. (a−f) Immunofluorescence staining and cellular fractionation assays of cells treated with NAC or Met to detect PKCζ nuclear translocation and LKB1 phosphorylation. FaDu cells were treated with or without NAC (10 mmol/L, and hereafter) for 10 h, followed by culture in GS condition in the presence or absence of NAC for an additional 2 h (a and e), or were treated with or without Met (10 mmmol/L, and hereafter) in the presence or absence of NAC under 4.5 mg/mL glucose condition for 12 h (c and f). Cells were subjected to immunofluorescence staining analyses (a and c) or western blot analyses (e and f). The co-localization between PKCζ and DAPI was quantified and statistically analyzed (b and d). A total of 30 cells derived from three independent experiments were randomly chosen and subjected to quantification analyses. (g−i) Immunofluorescence staining and cell fractionation analyses of cells treated by H_2_O_2_. FaDu cells were treated with or without H_2_O_2_ (1 mmol/L) for 30 min. Cells were subjected to immunofluorescence staining analyses (g) or cell fractionation and western blot analyses (i). The co-localization between PKCζ and DAPI was quantified and statistically analyzed (h). A total of 30 cells derived from three independent experiments were randomly chosen and subjected to quantification analyses. (j−l) Western blot and immunofluorescence staining analyses of liver samples from mice treated with NAC and following fasting. The 6-week-old male C57BL/6 mice (*n* = 3/group) were i.p. injected with NAC (200 mg/kg) daily. On day 7 after injection, mice were fasted for 16 h before being sacrificed. Mouse liver samples were subjected to western blot analyses (j) or immunofluorescence staining analyses (k). Pearson’s correlation coefficient was used to qualify the co-localization of PKCζ and DAPI, using images derived from immunostained liver samples (l). Data were presented as mean ± SD (b, d, and h) or SEM (l). Comparisons were performed with one-way ANOVA with Tukey’s test (b, d, and l) and unpaired two-tailed Student’s *t*-test (h). Scale bar, 25 μm. pAMPK: phospho-AMPK (Thr172); pS428-LKB1: phospho-LKB1 (Ser428).

It has been shown that cytosolic PKCζ can be translocated to the nucleus to phosphorylate LKB1 at Ser428, resulting in LKB1 cytoplasmic translocation and activation of AMPK [6]. We therefore examined the effects of ROS on PKCζ nuclear translocation and phosphorylation of LKB1 at Ser428 upon energy stress. The results showed that glucose starvation or metformin treatment promoted PKCζ nuclear translocation, accompanied by increased Ser428 phosphorylation *in vitro*, which could be effectively inhibited by NAC treatment (Fig. 2a–f; Supplementary Fig. S3a). Conversely, H_2_O_2_ treatment could significantly facilitate PKCζ nuclear translocation, as evidenced by immunofluorescent and cellular fractionation assays (Fig. 2g–i). To dissect the role of ROS on PKCζ subcellular localization *in vivo*, we analyzed the PKCζ−LKB1−AMPK axis in mice treated with fasting or metformin. The results showed that fasting or metformin treatment promoted the nuclear translocation of PKCζ and the phosphorylation of LKB1 at Ser428 in the liver samples, both of which could be largely inhibited by NAC treatment (Figure 2j–l; Supplementary Fig. S3b−e). Together, these results indicate that energy stress-induced ROS promotes PKCζ nuclear translocation to facilitate the phosphorylation of LKB1 at Ser428, resulting in LKB1 cytoplasmic translocation and AMPK activation.

### Energy stress-induced ROS promotes S-glutathionylation of PKCζ in facilitating PKCζ−KPNA2 protein complex formation and PKCζ nuclear translocation

**Figure 3:**
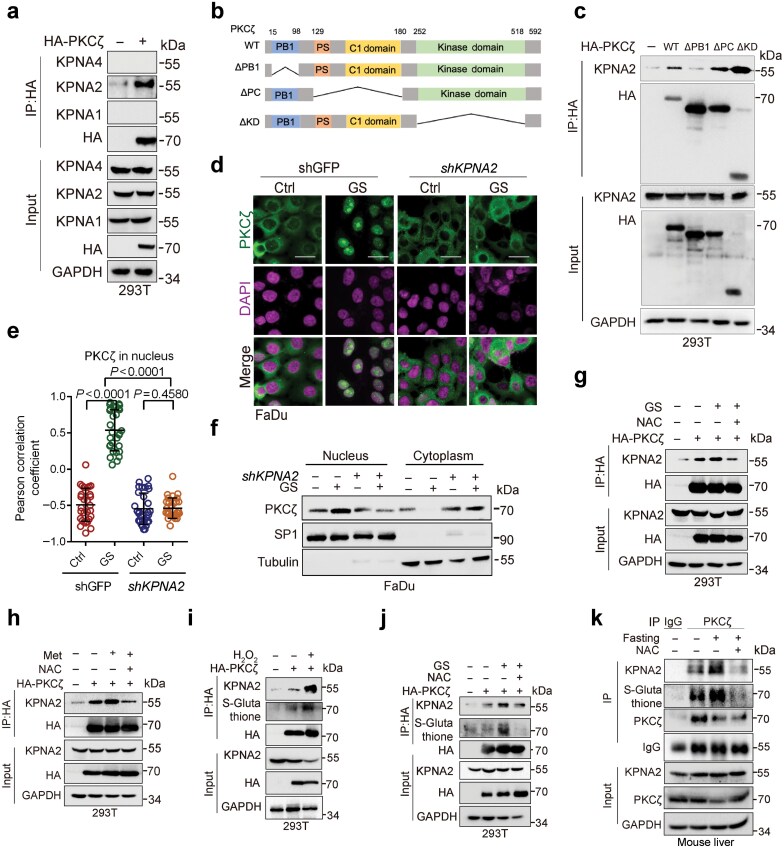
Energy stress-induced ROS promotes PKCζ S-glutathionylation to facilitate PKCζ−KPNA2 protein complex formation and PKCζ nuclear translocation. (a) Co-IP analyses of cells transiently expressing HA-PKCζ. 293T cells transiently expressing HA-PKCζ were subjected to Co-IP and western blot analyses. (b) A schematic diagram depicting the structural domains of the PKCζ protein. The diagram includes the following deletions: △PB1: deletion of the PB1 domain; △PC: deletion of the PS region to the C1 domain; △KD: deletion of the kinase domain. (c) Co-IP analyses of cells transfected with indicated plasmids. The 293T cells were transfected with indicated plasmids for 24 h, followed by Co-IP and western blot analyses. (d−f) Immunofluorescence staining analyses of cells stably expressing *shKPNA2* or shGFP grown under GS condition. FaDu cells stably expressing *shKPNA2* or shGFP were grown in GS condition for 2 h. Cells were subjected to immunofluorescence staining analyses (d) or were subjected to cell fractionation and western blot analyses (f). The co-localization between PKCζ and DAPI was quantified and statistically analyzed (e). A total of 30 cells derived from three independent experiments were randomly chosen and subjected to quantification analyses. (g) Co-IP analyses of cells transiently expressing HA-PKCζ treated with NAC and following culture in GS condition. The 293T cells transiently expressing HA-PKCζ were treated with or without NAC (10 mmol/L) for 10 h, followed by culture in GS condition in the presence or absence of NAC for an additional 2 h. Cells were then subjected to Co-IP and western blot analyses. (h) Co-IP analyses of cells transiently expressing HA-PKCζ treated with Met and NAC. The 293T cells transiently expressing HA-PKCζ were treated with or without Met (10 mmol/L) in the presence or absence of NAC for 12 h, followed by Co-IP and western blot analyses. (i) Co-IP analyses of cells transiently expressing HA-PKCζ treated with H_2_O_2_. The 293T cells transiently expressing HA-PKCζ were treated with or without H_2_O_2_ (1 mmol/L) for 30 min. Cells were then subjected to Co-IP and western blot analyses. (j) Co-IP analyses of cells transiently expressing HA-PKCζ treated with NAC and following culture in GS condition and additional 2-h NAC treatment. The 293T cells transiently expressing HA-PKCζ were treated with or without NAC for 10 h, followed by culture in GS condition in the presence or absence of NAC for an additional 2 h. Cells were then subjected to Co-IP and western blot analyses. (k) Co-IP analyses of liver samples from mice treated with NAC and following fasting. Six-week-old male C57BL/6 mice were i.p. injected with NAC (200 mg/kg) daily. On day 7 after injection, mice were fasted for 16 h before being sacrificed. Mouse liver samples were subjected to Co-IP and western blot analyses. Data were presented as mean ± SD (e). Comparisons were performed with one-way ANOVA with Tukey’s test (e). Scale bar, 25 μm.

We further investigated the molecular mechanisms by which energy stress-induced ROS promotes PKCζ nuclear translocation. The importin α/β family proteins are recognized as key regulators for the nuclear import of proteins harboring classical nuclear localization signals (NLSs) [20]. Notably, the analyses of the cNLS Mapper database (nls-mapper.iab.keio.ac.jp/cgi-bin/NLS_Mapper_help.cgi) showed that PKCζ bears a putative NLS sequence (Supplementary Fig. S4a). Therefore, we speculated that PKCζ may interact with importin α/β to facilitate PKCζ nuclear translocation. Co-immunoprecipitation (Co-IP) analyses showed that PKCζ could form stable protein complexes with KPNA2, but not with KPNA1 (importin α5) or KPNA4 (importin α3) (Fig. 3a). Further analyses showed that the Phox/Bem domain 1 (PB1) of PKCζ was critical for its interaction with KPNA2 (Fig. 3b and c). Notably, silencing of KPNA2 significantly inhibited glucose starvation-induced PKCζ nuclear translocation (Fig. 3d–f; Supplementary Fig. S4b). Furthermore, glucose starvation or metformin treatment promoted PKCζ−KPNA2 protein complex formation, which could be inhibited by NAC treatment (Fig. 3g and h).

We then explored the molecular basis by which ROS-mediated PKCζ−KPNA2 protein complex formation occurs. It has been established that redox modifications of cysteine (thiol) residues can result in reversible or irreversible alterations in protein structure and function, including disulfide bond formation (S-S), S-glutathionylation (S-S-G), sulfenylation (SOH), sulfinic acid (SO_2_H), and sulfonic acid (SO_3_H) [10]. To investigate what specific modification on PKCζ protein can impact PKCζ−KPNA2 protein complex formation, we first examined the effects of H_2_O_2_ on PKCζ protein modifications. Notably, S-glutathionylation of the PKCζ protein could be readily detected upon H_2_O_2_ treatment (Fig. 3i). In addition, glutathionylated PKCζ exhibited higher binding ability with KPNA2 (Fig. 3i). Further study showed that glucose starvation did not promote disulfide bond formation (Supplementary Fig. S4c). By contrast, glucose starvation promoted PKCζ S-glutathionylation and PKCζ−KPNA2 complex formation, both of which were largely suppressed by NAC treatment (Fig. 3j). We then investigated the effects of ROS on PKCζ S-glutathionylation and PKCζ−KPNA2 complex formation *in vivo*. As shown in Fig. 3k, fasting of mice led to increased PKCζ S-glutathionylation concomitant with enhanced PKCζ−KPNA2 complex formation in the liver, which could be effectively suppressed by NAC treatment.

Together, these results indicate that ROS is pivotal in energy stress-induced PKCζ S-glutathionylation to facilitate PKCζ−KPNA2 protein complex formation and PKCζ nuclear translocation.

### Cys48-S-glutathionylation of PKCζ is critical for PKCζ−KPNA2 protein complex formation and PKCζ nuclear translocation

**Figure 4:**
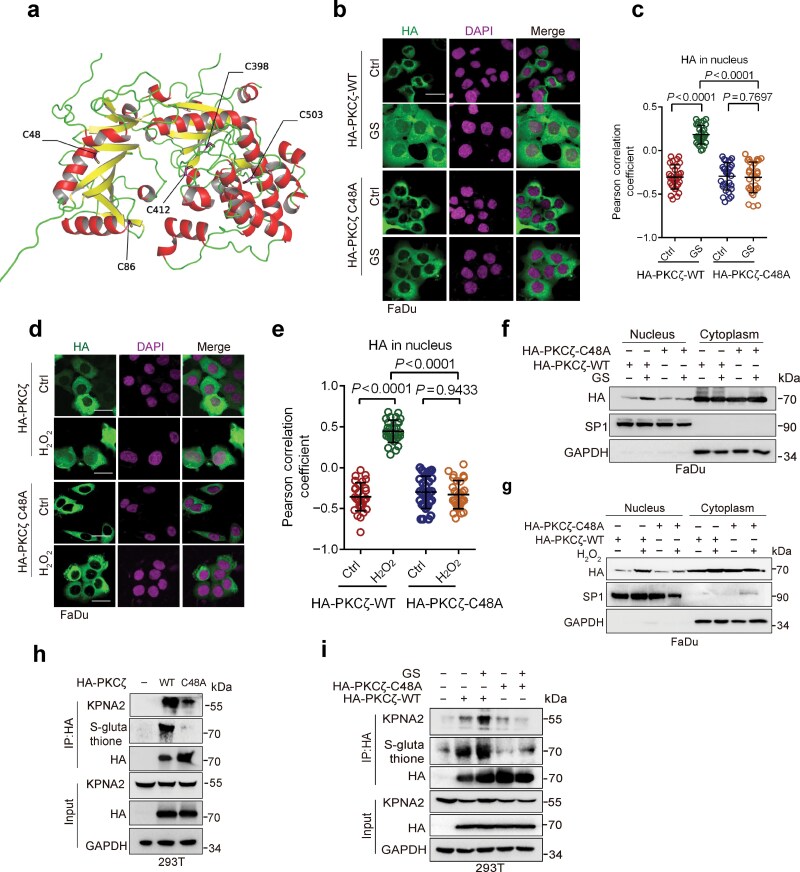
PKCζ S-glutathionylation at Cys48 facilitates PKCζ−KPNA2 protein complex formation and PKCζ nuclear translocation. (a) The projected structure of PKCζ obtained from the Alphafold2 analyses [21]. (b−g) Immunofluorescence staining and fractionation analyses of FaDu cells stably expressing wild-type HA-PKCζ (HA-PKCζ-WT) or HA-PKCζ-C48A that were grown in GS condition or treated with H_2_O_2_. FaDu cells stably expressing HA-PKCζ-WT or HA-PKCζ-C48A were grown in GS condition for 2 h (b and f), or were treated with or without H_2_O_2_ (1 mmol/L) for 30 min (d and g). Cells were subjected to immunofluorescence staining analyses (b and d) or were subjected to fractionation and western blot analyses (f and g). The co-localization between PKCζ and DAPI was quantified and statistically analyzed (c and e). A total of 30 cells derived from three independent experiments were randomly chosen and subjected to quantification analyses. (h) Co-IP analyses of 293T cells transiently expressing HA-PKCζ-WT or HA-PKCζ-C48A. The 293T cells transiently expressing HA-PKCζ-WT or HA-PKCζ-C48A were subjected to Co-IP and western blot analyses. (i) Co-IP analyses of 293T cells grown in GS condition. The 293T cells transiently expressing HA-PKCζ-WT or HA-PKCζ-C48A were grown in GS condition for 2h. Cells were then subjected to Co-IP and western blot analyses. Data were presented as mean ± SD (c and e). Comparisons were performed with one-way ANOVA with Tukey’s test (c and e). Scale bar, 25 μm.

To further investigate which cysteine residue(s) of PKCζ was glutathionylated upon energy stress, we performed the Oximouse database analyses (oximouse.hms.harvard.edu/sites.html), which revealed that five cysteine residues (Cys48, Cys86, Cys412, Cys398, and Cys502) could be oxidized in the heart tissue of aged mice (Fig. 4a). We then examined a series of mutant proteins bearing either C48A, C86A, C398A, C412A, or C503A, in their ability to undergo nuclear translocation upon energy stress. Similar to wild-type PKCζ, PKCζ-C86A, PKCζ-C398A, PKCζ-C412A, and PKCζ-C503A, but not PKCζ-C48A, were able to translocate into the nucleus upon glucose starvation or H_2_O_2_ treatment, as evidenced by immunofluorescence staining (Fig. 4b–e; Supplementary Fig. S5a and b). Consistently, cellular fractionation analyses showed that either glucose starvation or H_2_O_2_ treatment also facilitated nuclear translocation of wild-type PKCζ, but not PKCζ-C48A (Fig. 4f and g).

To substantiate the importance of S-glutathionylation of PKCζ at Cys48, we performed immunoprecipitation analyses. Compared to wild-type PKCζ, the PKCζ-C48A exhibited significantly reduced S-glutathionylation and interaction with KPNA2 (Fig. 4h), indicating that S-glutathionylation at the C48 residue of PKCζ is critical for KPNA2 interaction. Consistently, glucose starvation significantly increased S-glutathionylation of wild-type PKCζ and more PKCζ−KPNA2 complex formation but had little effect on PKCζ-C48A (Fig. 4i). Together, these results indicate that energy stress-induced ROS promotes S-glutathionylation of PKCζ at Cys48 to facilitate PKCζ−KPNA2 protein complex formation and PKCζ nuclear localization.

### Diminished ROS levels exacerbate high-fat diet (HFD)-induced lipid accumulation in mouse liver and impair the hypoglycemic effectiveness of metformin

**Figure 5:**
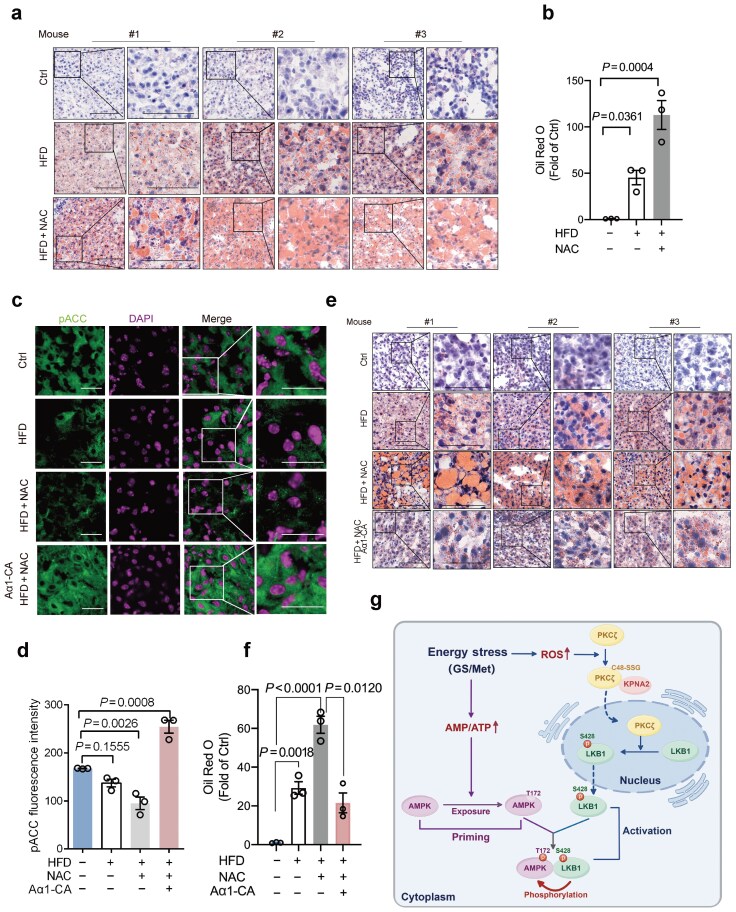
Diminished ROS levels exacerbates the HFD-induced lipid accumulation in mouse livers. (a and b) Oil Red O staining analysis of livers from mice treated with NAC upon HFD. The 6-week-old male C57BL/6 mice were i.p. injected with or without NAC (200 mg/kg) daily upon HFD. On day 21 after injection, mice were sacrificed and mouse livers were subjected to Oil Red O staining (a). The lipid levels were quantified and statistically analyzed (b). (c−f) Immunofluorescence staining and Oil Red O staining analyses of livers overexpressing Aα1-CA with NAC treatment upon HFD. The 6-week-old male C57BL/6 mice were infected with 2 × 10^7^ PFU lentivirus carrying an AMPKα1 constitutively active mutant (Aα1-CA) by tail-vein injection. On day 3 after injection, mice were i.p. injected with NAC (200 mg/kg) daily upon HFD. On day 21 after the NAC injection, mice were sacrificed and mouse livers were subjected to immunofluorescence staining (c and d) or Oil Red O staining (e). The lipid levels were quantified and statistically analyzed (f). (g) A model depicting that ROS-mediated PKCζ S-glutathionylation at Cys48 is essential for LKB1 cytoplasmic localization and AMPK activation upon energy stress. SSG: S-glutathionylation. Data were presented as mean ± SEM (b, d, and f). Comparisons were performed with one-way ANOVA with Tukey’s test (b, d, and f). Scale bar, 25 μm.

Dysfunction of AMPK is associated with various human metabolic disorders, including fatty liver, diabetes, obesity, and the development of cancers [19, 22]. To explore the role of ROS-induced PKCζ S-glutathionylation and AMPK activation under a pathophysiological setting, we examined the effects of HFD-induced lipid accumulation in mouse livers. The results showed that HFD significantly induced lipid accumulation in mouse livers (Fig. 5a and b), in keeping with a previous report [23]. Notably, lipid accumulation was dramatically increased in the livers derived from mice simultaneously treated with HFD and NAC. To investigate the causal role of ROS-induced S-glutathionylated PKCζ−AMPK axis in HFD-induced lipid accumulation, we employed a tail-vein injection of recombinant lentiviruses expressing a constitutively active AMPKα1 (Aα1-CA), whose kinase activity was not affected by ROS levels (Fig. 5c and d; Supplementary Fig. S2d). The results showed that while NAC treatment substantially augmented lipid accumulation in the livers of HFD-fed mice, NAC had only marginal effects on HFD-induced lipid accumulation in the liver with ectopic overexpression of Aα1-CA (Fig. 5e and f).

Metformin is known to decrease blood glucose levels through the activation of AMPK [24]. Given that ROS is important in metformin-mediated AMPK activation, we investigated the effects of ROS reduction on the metformin-induced hypoglycemic efficacy in a type 2 diabetic mouse model induced by streptozotocin (STZ) (Supplementary Fig. S6a−d). In keeping with previous findings [25], metformin treatment led to a significant reduction in blood glucose levels in this mouse model, which was completely abrogated by NAC treatment (Supplementary Fig. S6e), indicating that disruption of ROS homeostasis results in the inactivation of AMPK, concomitant with impeding the metformin efficacy in lowering blood glucose levels.

Collectively, these results indicate that disruption of ROS homeostasis not only impedes AMPK activity but also exacerbates lipid accumulation in mouse livers and reduces the hypoglycemic efficacy of metformin.

## Discussion

Abundant evidence indicates that LKB1-mediated AMPK activation involves two critical steps: (i) Priming—the exposure of Thr172 on AMPKα, and (ⅱ) activating—the cytoplasmic LKB1-induced Thr172 phosphorylation of AMPKα. Energy deprivation leads to an increase of AMP that binds to the α-subunit of AMPK, resulting in exposing Thr172 of AMPKα [2]. Yet, the precise mechanism triggering LKB1 nuclear export in response to energy stress remains elusive. Here we demonstrate that energy stress leads to the elevation of ROS that triggers S-glutathionylation of PKCζ at Cys48 to facilitate PKCζ−KPNA2 protein complex formation and PKCζ nuclear translocation, which in turn phosphorylates LKB1 at Ser428, consequently resulting in LKB1 cytoplasmic translocation (Fig. 5g). Therefore, this study reveals that the activation of the ROS−PKCζ−KPNA2−LKB1 axis is a pivotal activating process for AMPK activation upon energy stress. However, it is reported that glucose starvation may not always elevate intracellular ROS levels [26]. Moreover, in 293T and MEF (mouse embryonic fibroblast) cells, reducing ROS only partially suppresses AMPK activation induced by glucose deprivation or metformin treatment. These observations suggest that, in certain contexts, AMPK may also be activated via alternative, ROS-independent mechanisms, such as calcium signalling [27] or depletion of glycolytic intermediates like fructose-1,6-bisphosphate [5].

In addition to S428, phosphorylation of LKB1 at S307 and T189 also plays a crucial role in promoting its cytoplasmic translocation and subsequent AMPK activation [28, 29]. Consistent with this, our results show that both wild-type LKB1 and the S428A mutant could induce AMPK activation, indicating that LKB1 was critical for AMPK activation at the basal level (Supplementary Fig. S7a). Under glucose starvation, wild-type LKB1 induced AMPK activation more potently than the S428A mutant (Supplementary Fig. S7b). These findings suggest that phosphorylation at S428 is crucial but not entirely necessary for glucose starvation-induced AMPK activation. Our study identifies the ROS−PKCζ−LKB1-S428 axis as a novel pathway through which energy stress induces LKB1 cytoplasmic translocation and AMPK activation. However, whether phosphorylation at other LKB1 sites, such as S307 and T189, also contributes to this process remains to be further explored.

It has been reported that ROS plays a critical role in the activation of AMPK, but the precise mechanism remains debatable. On one hand, ROS has been reported to directly oxidize and activate AMPK [30]. On the other hand, a study reported that ROS does not act directly on AMPK itself but indirectly modulates AMPK activity by altering the cellular AMP/ATP ratio [31]. Here, we show that ROS promotes the nuclear import of PKCζ and cytoplasmic translocation of LKB1 to promote phosphorylation and activation of AMPK, thereby unveiling an additional layer in the regulatory role of ROS on AMPK activity. Given the fact that persistent elevation of ROS is often harmful to cell physiology, it is plausible that maintaining cellular ROS homeostasis is critical for normal physiology. Indeed, activation of AMPK is pivotal in maintaining NADPH homeostasis to reduce ROS for tumor cell survival in response to energy stress [32]. Therefore, the ROS−AMPK−NADPH may form a feedback loop important for ROS homeostasis.

PKCζ is predominantly localized in the cytosol [6]. Several reports showed that PKCζ can translocate from the cytosol to the nucleus. For instance, ischemia has been shown to promote PKCζ nuclear translocation in Langendorff-perfused rat hearts [33]. H_2_O_2_ can trigger nuclear translocation of PKCζ to enhance cancer cell chemoresistance [34]. However, the precise mechanism(s) by which PKCζ translocates to the nucleus remains unknown. Here, we demonstrate that ROS-mediated S-glutathionylation of PKCζ at Cys48 enhances its interaction with KPNA2 (importin α2), thereby facilitating PKCζ nuclear import. These findings provide a mechanistic explanation for PKCζ nuclear translocation.

In addition to PKCζ, as shown in this study, several reports highlight the significance of S-glutathionylation in the nuclear translocation of proteins, including GAPDH and FABP5 [13, 14], raising a possibility that S-glutathionylation and subsequent interaction might be a common mechanism for protein nuclear import. Notably, glutaredoxin 1 (Grx1) is known to catalyze S-glutathionylation of various proteins, including GAPDH, protein tyrosine phosphatase 1B (PTP1B), p65, and actin [35]. Given that Grx1 plays a key role in cell survival during glucose deprivation in neurons and cancer cells [36, 37], it is plausible that Grx1-induced S-glutathionylation of PKCζ protein and subsequent activation of AMPK play a key role in cell survival under energy stress.

AMPK is a central player in regulating energy homeostasis. Disruption of AMPK function leads to several human diseases [22]. Our previous studies demonstrate that oncogenic signaling, including Ras, phosphatidylinositol 3-kinase (PI3K), or human epidermal growth factor receptor 2 (HER2), can suppress AMPK transcription, leading to the disruption of cell−cell adhesion in promoting breast cancer metastasis [19]. In this study, we established the criticality of the ROS−PKCζ−KPNA2 axis for AMPK activation, suggesting that perturbations in this axis might be implicated in metabolic disorders. Indeed, diminished PKCζ activity is associated with insulin resistance in obese and type 2 diabetic individuals [38]. Notably, our results indicate that disruption of ROS homeostasis leads to a deficiency of AMPK activation accompanied by lipid accumulation in mouse livers as well as impeding the metformin efficacy in lowering blood glucose levels of mouse models, suggesting that overdosing of antioxidants, such as excess of dietary supplements that may perturb ROS homeostasis, might need to be carefully reevaluated.

However, the role of AMPK in fatty liver remains controversial. One study reported that AMPK deletion does not exacerbate HFD-induced fatty liver formation [39], whereas another showed that AMPK deficiency promotes HFD-induced hepatic lipid accumulation [40]. In contrast, several studies have demonstrated that AMPK activation suppresses HFD-induced hepatic lipid accumulation [39, 41–43], highlighting the complex and context-dependent role of AMPK in hepatic lipid metabolism. In this study, we show that ROS scavenging by NAC enhances HFD-induced hepatic lipid accumulation, accompanied by reduced AMPK activity. Notably, reactivation of AMPK significantly attenuates lipid accumulation in livers from HFD + NAC-treated mice. These findings underscore a critical role for the ROS−AMPK axis in regulating hepatic lipid homeostasis. However, whether NAC accelerates long-term progression of fatty liver disease remains to be determined.

### Limitations of the study

This study investigates the role of S-glutathionylation of PKCζ at Cys48 in mediating LKB1 cytoplasmic translocation and subsequent AMPK activation under energy stress. However, the lack of *in vivo* validation limits the conclusions regarding the physiological significance of this modification in AMPK regulation. Moreover, whether S-glutathionylation of PKCζ at Cys48 contributes to the development or progression of metabolic diseases, such as obesity, non-alcoholic fatty liver disease, and type 2 diabetes, remains unknown and warrants further investigation.

## Materials and methods

### Cell culture and reagents

FaDu (HTB-43) and 293T (CRL-11268) cells were obtained from ATCC (Manassas, VA, USA). HEK-293FT (R70007) cells were obtained from Thermo Fisher Scientific (Waltham, MA, USA). A549 (BNCC337696) cells were obtained from the BeNa Culture Collection (Beijing, China). MEF cells were isolated from day 13.5 mouse embryos. All cell lines used in this study were routinely tested to be negative for mycoplasma contamination and were kept at low passages to maintain their identity. FaDu, MEF, 293T, HEK-293FT, and A549 cells were cultured in DMEM medium (Gibco, Rockville, MD, USA) supplemented with 10% fetal bovine serum (FBS; HyClone, Logan, UT, USA), 100 units/mL penicillin (Gibco, Rockville, MD, USA), and 100 μg/mL streptomycin (Gibco, Rockville, MD, USA). Cells were grown in a humidified 37°C incubator in 5% CO_2_ atmosphere. Cells at 60%−70% confluence were treated with an indicated chemical compound. NAC (A9165) was purchased from Sigma-Aldrich (St. Louis, USA). Metformin (A506198) was purchased from Sangon Biotech (Shanghai, China). H_2_O_2_ (7722) was purchased from Chron Chemicals (Chengdu, China). Dithiothreitol (DTT, R0861) was purchased from Selleck Chemicals (Houston, USA). Vitamin E (S0079) and glutathione (GSH, S0073) were purchased from Beyotime (Shanghai, China). A-23187(HY-N6687) was purchased from MCE (Shanghai, China).

### Plasmid transfection, lentiviral infection, and RNA interference

Cells at 70% confluence were transfected using Lipofectamine 2000 (Invitrogen, Carlsbad, CA, USA). Expression plasmids used in this study included human Flag-AMPKα1-WT, Flag-AMPKα1-CA, Flag-LKB1, Flag-HA-PKCζ-WT, HA-PKCζ-C48A, HA-PKCζ-C86A, HA-PKCζ-C398A, HA-PKCζ-C412A, and HA-PKCζ-C503A. Recombinant lentiviruses were amplified by transfection of HEK-293FT cells with pMD2.G and psPAX2 packaging plasmids and lentiviral expression plasmid using Lipofectamine 2000. Viruses were collected 60 h after transfection. Cells at 65% confluence in the presence of 10 μg/mL polybrene were infected with recombinant lentivirus, followed by 12 h of incubation at 37°C with 5% CO_2_. Lentivirus-based shRNAs targeting *KPNA2* (CCTGGACACTTTCTAATCTTT) or green fluorescent protein (GAAGCAGCACGACTTCTTC) were constructed into a pLKO.1-puromycin lentiviral vector.

### Cellular fractionation

Cellular fractionation of cytoplasm and nucleus was performed as described in the Nuclear and Cytoplasmic Protein Extraction Kit (P0028, Beyotime, Chengdu, China). Briefly, cells were collected, washed twice with cold PBS, and resuspended in cytosolic lysis buffer. The samples were vortexed vigorously at the highest speed for 5 s to completely suspend and disperse the cell precipitate, and then ice bathed for 10−15 min. After centrifugation, the cytoplasm fraction in the supernatant was collected. Then nuclear lysis buffer was added to the precipitation, and the samples were vortexed strongly for 30 min. The samples were then centrifuged and the nuclear fraction was collected.

### Measurement of ROS levels

Cells (2 × 10^5^) were seeded per well in 24-well plates and grown overnight, replaced with fresh regular DMEM in the absence or presence of indicated chemical compound(s) as designed. Cells were washed and subjected to the procedures as described in the Reactive Oxygen Species Assay Kit (S0033, Beyotime). Totally, 2 × 10^4^ cells were analyzed by fluorescence-activated cell sorting (FACS) and presented accordingly.

### Western blot, Co-IP, and immunofluorescence staining analyses

For western blot analysis, cells were collected, washed twice with cold PBS, and lysed in 1 × SDS Sample Buffer (#7722, Cell Signaling Technology, USA) according to the manufacturer’s protocol supplemented with proteasome inhibitor cocktail. Equal amounts of protein were loaded, separated by SDS-PAGE, and transferred to PVDF membranes (Millipore, Darmstadt, Germany). Membranes were blocked in 5% non-fat dry milk and hybridized to primary antibodies and horseradish peroxidase (HRP)-conjugated secondary antibody for subsequent detection by chemiluminescence (Bio-Rad ChemiDoc XRS+, Bio-Rad). Gel and blot images were analyzed using Image Lab Software 5.0. Antibodies for GAPDH (P8833, 1:3000), SP1 (SC59, 1:1000), and Tubulin (sc58886, 1:1000) were purchased from Santa Cruz (USA). Antibodies for pAMPK (#2535, 1:1000), pACC (#3661,1:1000), LKB1 (#3050, 1:1000), pLKB1 (#3482, 1:1000), HA (#3724, 1:1000), and Flag (#117935, 1:1000) were purchased from Cell Signaling Technology (Danvers, MA, USA). Antibody for PKCζ (R26340) was purchased from Zenbio (Chengdu, China). Anti-glutathione (ab19534, 1:1000) was purchased from Abcam (Cambridge, MA, USA). Antibodies for KPNA2 (ET1705-61, 1:1000) and KPNA4 (ER1912-10, 1:1000) were purchased from Huabio (Hangzhou, China). Antibody for KPNA1 (A1742, 1:1000) was purchased from Abclonal (Wuhan, China). Antibody for Histone 3 (H3, CY6587, 1:1000) was purchased from Abways (Shanghai, China).

For endogenous Co-IP, cells were lysed in IP buffer (1 mmol/L Tris, pH 7.5, 5 mmol/L NaCl, 0.25% Nonidet P-40, 0.1% deoxycholate, and proteasome inhibitors). Equal amounts of total protein were incubated with primary antibodies or normal indicated IgG overnight at 4°C, and then 30 µL of protein A/G beads were added for an additional 2 h of incubation. For exogenous Co-IP, anti-HA beads or anti-Flag beads were added to equal amounts of total protein and incubated overnight. Beads were centrifuged (500 *g* for 30 s) and washed three times using wash buffer (20 mmol/L Tris-HCl, 250 mmol/L NaCl, 0.2 mmol/L EGTA, and 0.1% Nonidet P-40). The beads were heated at 100°C for 10 min before western blot analysis. Anti-FLAG M2 affinity gel (A2220) was purchased from Sigma-Aldrich (St. Louis, USA). Pierce Anti-HA magnetic beads (#88836) were purchased from Thermo Fisher Scientific (Waltham, MA, USA).

For Immunofluorescence staining, cells grown on coverslips were fixed with 4% polyformaldehyde in PBS, permeabilized with 0.1% Triton X-100 in PBS, blocked with 4% bovine serum albumin in PBS, and hybridized to an appropriate primary antibody (HA-Tag, 1:1000, #3724, CST), followed by incubation with a second antibody (Goat anti-Mouse Alexa Fluor 488, A-11029 or Goat anti-Rabbit Alexa Fluor 514, A-31558, ThermoFisher). The cells were counterstained with ProLong® Gold Antifade Reagent with DAPI (#82961, CST) before visualization and photographed using a Leica TCS SP5II confocal laser scanning microscope. LAS X (V3.3.0) was used to analyze fluorescent images. To determine the co-localization of PKCζ or LKB1 with DAPI, the free software Image J/Fiji, coupled with the Coloc 2 plugin and Pearson’s correlation coefficient, was used to calculate double fluorescence correlation coefficients (Ref), and co-localized fluorescence quantifications were presented by scatter diagrams.

### Mouse models

Male C57BL/6 mice were purchased from GemPharmatech (Chengdu, China). Mice were maintained in individual cages at room temperature of (22 ± 2)°C and humidity of 50%–60%, on a 12-h light/12-h dark cycle (lights on at 09:00). Six-week-old male C57BL/6 mice were intraperitoneally (i.p.) injected with NAC (200 mg/kg) and/or metformin (50 mg/kg), or mice were fasted for an indicated time point. Mice were sacrificed, and mouse liver samples were subjected to immunofluorescence staining and western blot analyses.

### Statistics and reproducibility

GraphPad Prism 8.0 (GraphPad Software Inc., USA) was used for data recording, collection, processing, and calculation. Data from at least three independent experiments *in vitro* were presented as mean ± standard deviation (SD), and data from animal experiments were presented as mean ± standard error of the mean (SEM). An unpaired two-tailed Student’s *t*-test was used for comparing two groups of data. One-way ANOVA with Tukey’s test was used to compare multiple groups of data. *P* values ≤ 0.05 were considered to be significant.

## Supplementary Material

loaf027_suppl_Supplementary_Figures_S1-S7

## Data Availability

The authors confirm that all the data supporting the findings of this study are available within the supplementary material and corresponding authors.
