# Supplementary material for: Energy stress-induced PKCζ S-glutathionylation is essential for LKB1 cytoplasmic translocation and AMPK activation
Source: Life Metab. 2025 Jul 16;4(6):loaf027. doi: 10.1093/lifemeta/loaf027 (PMC12507020; doi:10.1093/lifemeta/loaf027)
Supplement: loaf027_suppl_Supplementary_Figures_S1-S7 [file loaf027_suppl_supplementary_figures_s1-s7.docx]

**Supplementary Information for**

**Energy stress-induced** **PKCζ S-glutathionylation is essential for**

**LKB1 cytoplasmic translocation and AMPK activation**

Junjie Fei^1^, Shuhan Yu^1^, Mingzhe Xu^1^, Yang Liu^1^, Guoqiang Wang^1^, Xueqing Li^1^, Xinyue Yu^1^, Yifan Zhang^1^, Wenhua Zhang^1^, Yang Wang^1^, Mengmeng Niu^1^, Yujun Zhang^1^, Yang Cao^1,*^, Zhi-Xiong Jim Xiao^1,2,*,†^, Yong Yi^1,*^

**Affiliations**

^1^Center of Growth, Metabolism and Aging, Key Laboratory of Bio-Resource and Eco-Environment of Ministry of Education, College of Life Sciences, Sichuan University, Chengdu, Sichuan 610064, China

^2^State Key Laboratory of Biotherapy, West China Hospital, Sichuan University, Chengdu, Sichuan 610041, China

^*^Corresponding authors. Center of Growth, Metabolism and Aging, Key Laboratory of Bio-Resource and Eco-Environment of Ministry of Education, College of Life Sciences, Sichuan University, Chengdu, Sichuan 610064, China. E-mail: yy-yiyong@scu.edu.cn (Y.Y.); jimzx@scu.edu.cn (Z.X.J.X.); cao@scu.edu.cn (Y.C.)

^†^Lead contact.

**Supplementary Figures S1−7**


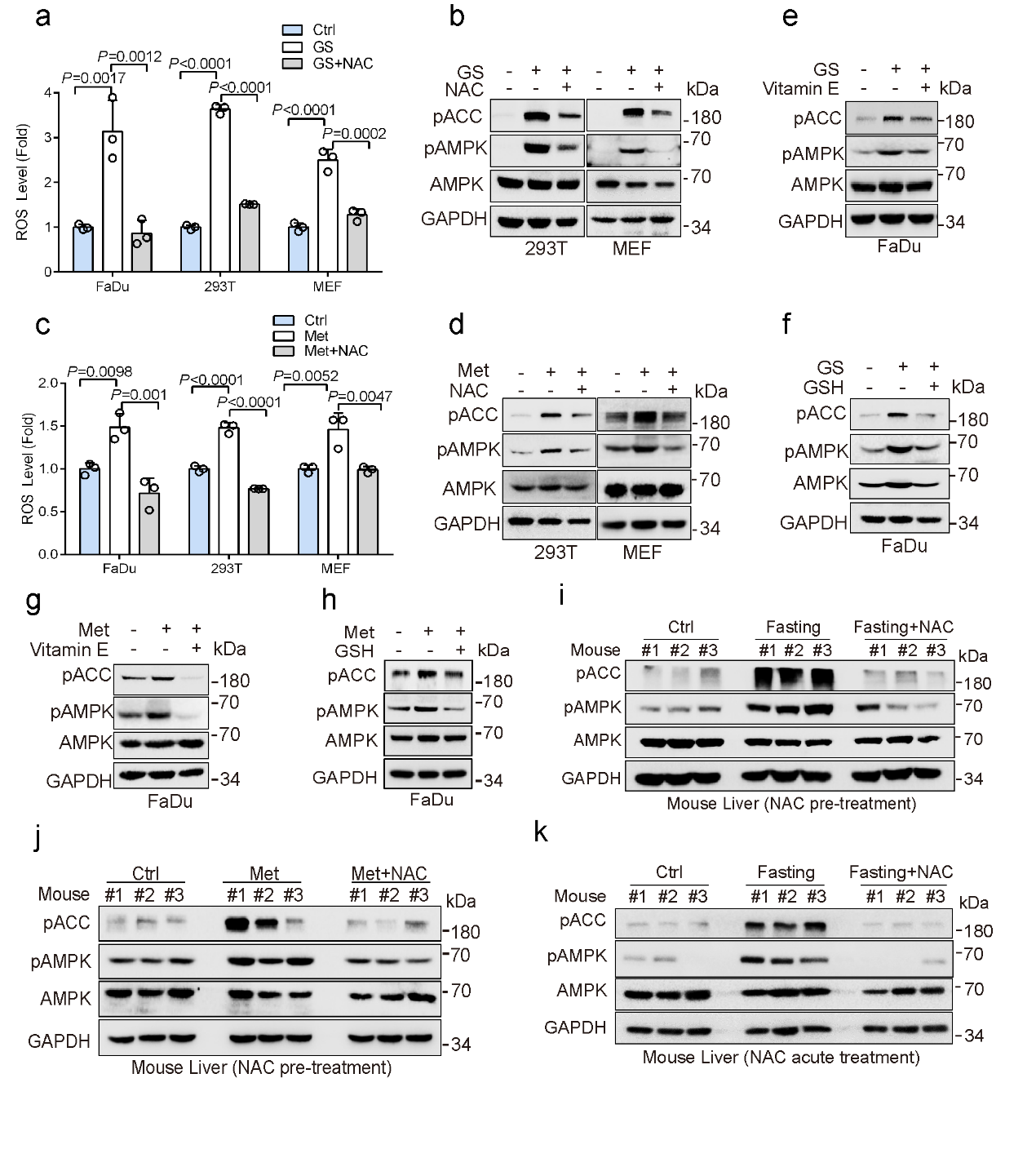


**Supplementary Figure S1** Reduction of ROS suppresses energy stress-induced activation of AMPK. (a−d) FaDu, 293T, or MEF cells were treated with or without N-Acetylcysteine (NAC, 10 mmol/L, and hereafter) for 10 h, followed by culture in 0 mg/mL glucose condition (glucose starvation, GS) in the presence or absence of NAC for an additional 2 h (a and b) or were treated with metformin (Met, 10 mmol/L, and hereafter) in the presence or absence of NAC under 4.5 mg/mL glucose condition for 12 h (c and d). Cells were subjected to measurement of ROS levels (a and c) or western blot analyses (b and d). (e and f) FaDu cells were treated with or without vitamin E (200 μmol/L, e) or glutathione (GSH, 1 mmol/L, f) for 10 h, followed by culture in GS condition in the presence or absence of vitamin E or GSH for additional 2 h. Cells were subjected to western blot analyses. (g and h) FaDu cells were treated with or without Met in the presence or absence of vitamin E (200 μmol/L, g) or GSH (1 mmol/L, h) under 4.5 mg/mL glucose condition for 12 h. Cells were subjected to western blot analyses. (i) The 6-week-old male C57BL/6 mice (*n* = 3/group) were intraperitoneally (i.p.) injected with NAC (200 mg/kg) daily (NAC pre-treatment). On day 7 after injection, mice were fasted for 16 h before being sacrificed. Mouse livers were subjected to western blot analyses. (j) The 6-week-old male C57BL/6 mice (*n* = 3/group) were i.p. injected with NAC (200 mg/kg) and/or Met (50 mg/kg) daily. On day 14 after injection, mice were sacrificed. Mouse livers were subjected to western blot analyses. (k) The 6-week-old male C57BL/6 mice (*n* = 3/group) were fasted and i.p. injected with NAC (200 mg/kg) for 16 h before being sacrificed (NAC acute treatment). Mouse livers were subjected to western blot analyses. Comparisons were performed with one-way ANOVA with Tukey’s test (a and c). pAMPK: phospho-AMPK (Thr172); pACC: phospho-ACC (Ser79).


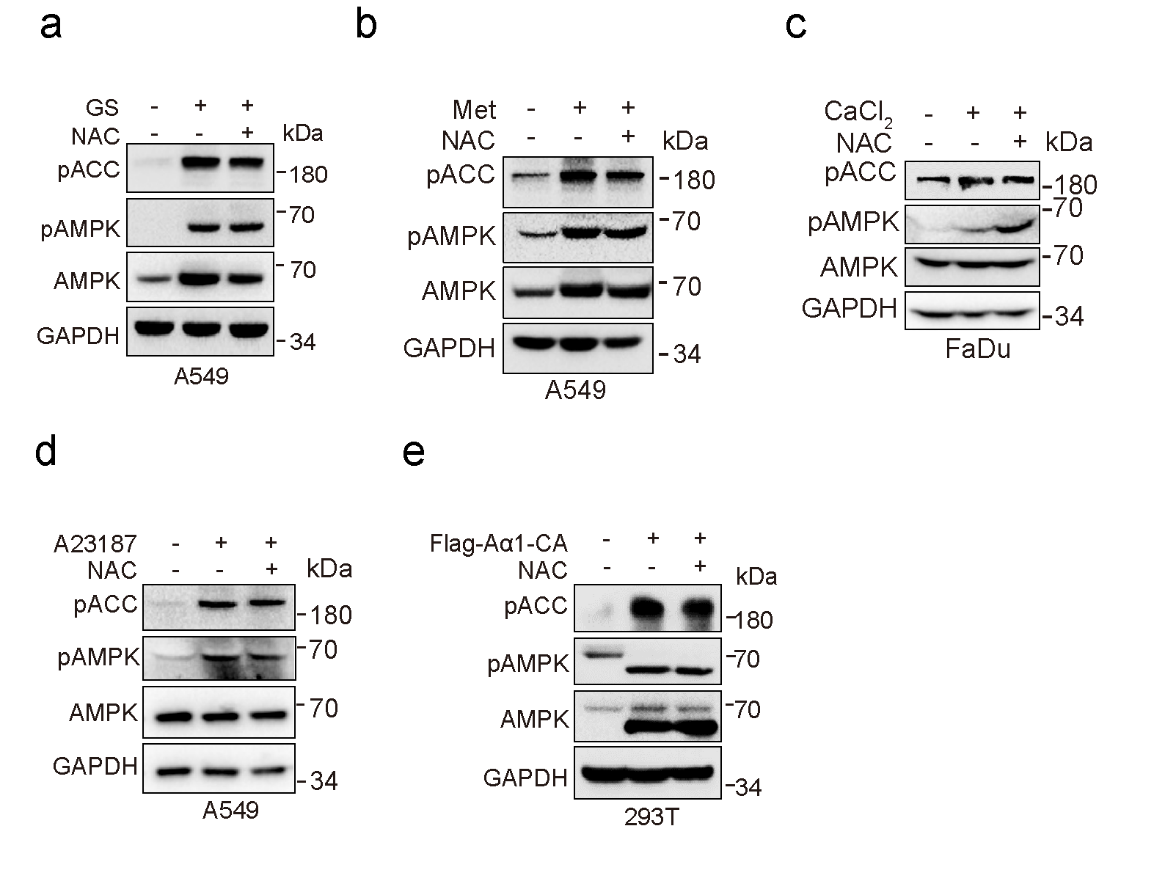


**Supplementary Figure S2** LKB1 is indispensable for ROS-mediated AMPK activation. (a) A549 cells were treated with or without NAC (10 mmol/L, and hereafter) for 10 h, followed by culture in GS condition in the presence or absence of NAC for an additional 2 h. Cells were subjected to western blot analyses. (b) A549 cells were treated with or without Met (10 mmol/L) under 4.5 mg/mL glucose condition in the presence or absence of NAC for 12 h. Cells were subjected to western blot analyses. (c) A549 cells were treated with or without CaCl_2_ (1 mmol/L) in the presence or absence of NAC for 12 h. Cells were subjected to western blot analyses. (d) A549 cells were treated with or without A23187 (0.5 μmol/L) in the presence or absence of NAC for 12 h. Cells were subjected to western blot analyses. (e) 293T cells expressing a constitutively active mutant, Flag-AMPKα1-T172D (Flag-Aα1-CA), were treated with or without NAC for 12 h. Cells were subjected to western blot analyses. pAMPK: phospho-AMPK (Thr172); pACC: phospho-ACC (Ser79).


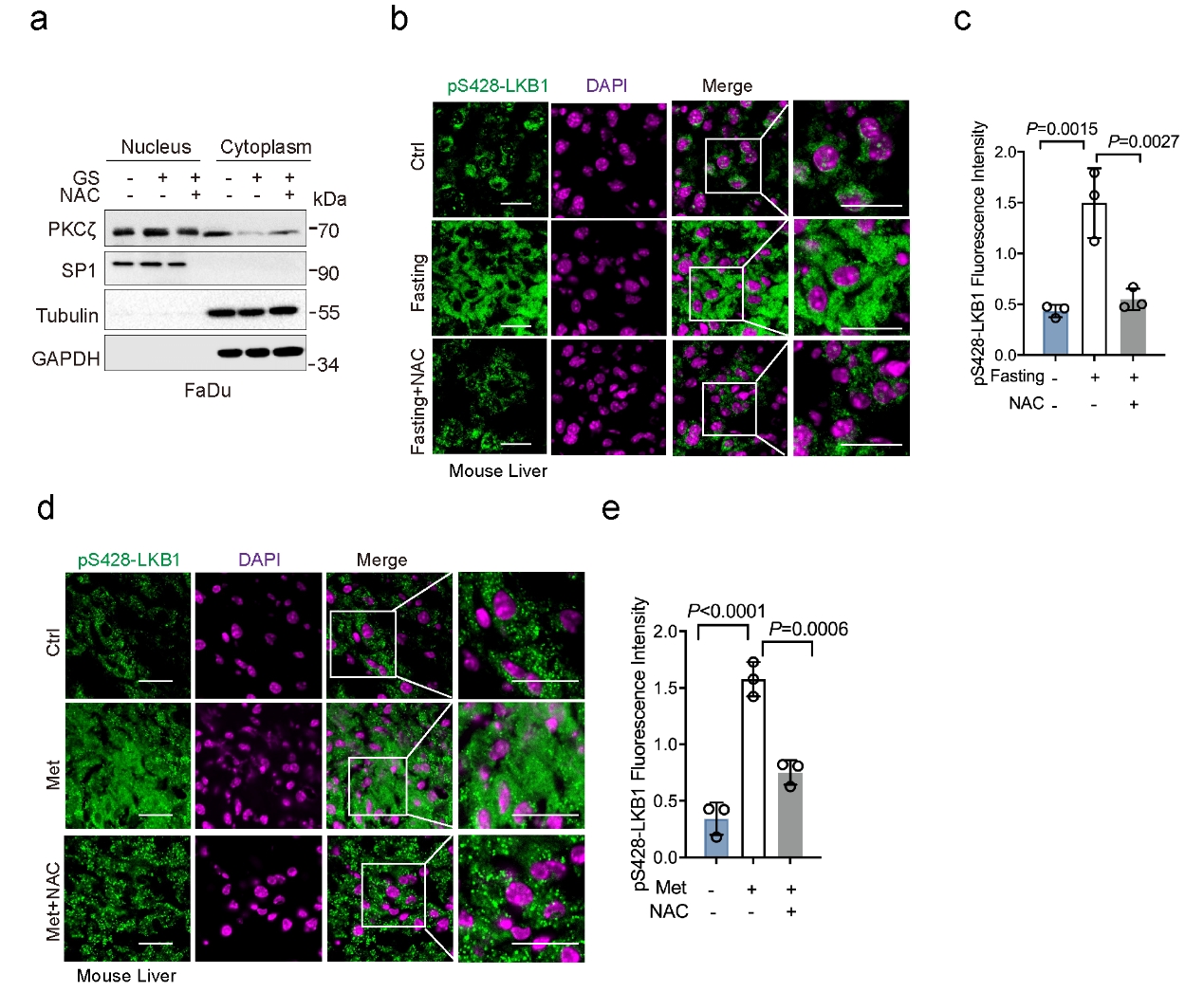


**Supplementary Figure S3** Energy stress-induced ROS is required for LKB1 phosphorylation at Ser428 and cytoplasmic translocation. (a) FaDu cells were treated with or without NAC (10 mmol/L) for 10 h, followed by culture in GS condition in the presence or absence of NAC for an additional 2 h. Cells were subjected to cell fractionation and western blot analyses. (b and c) The 6-week-old male C57BL/6 mice (*n* = 3/group) were i.p. injected with NAC (200 mg/kg) daily. On day 7 after injection, mice were fasted for 16 h before being sacrificed. Mouse liver sections were subjected to immunofluorescence staining analyses. (d and e) The 6-week-old male C57BL/6 mice (*n* = 3/group) were i.p. injected with NAC (200 mg/kg) and/or Met (50 mg/kg) daily. On day 14 after injection, mice were sacrificed. Mouse liver sections were subjected to immunofluorescence staining analyses. Data were presented as mean ± SEM (c and e). Comparisons were performed with one-way ANOVA with Tukey’s test (c and e). Scale bar, 25 μm. pS428-LKB1: phospho-LKB1 (Ser428).


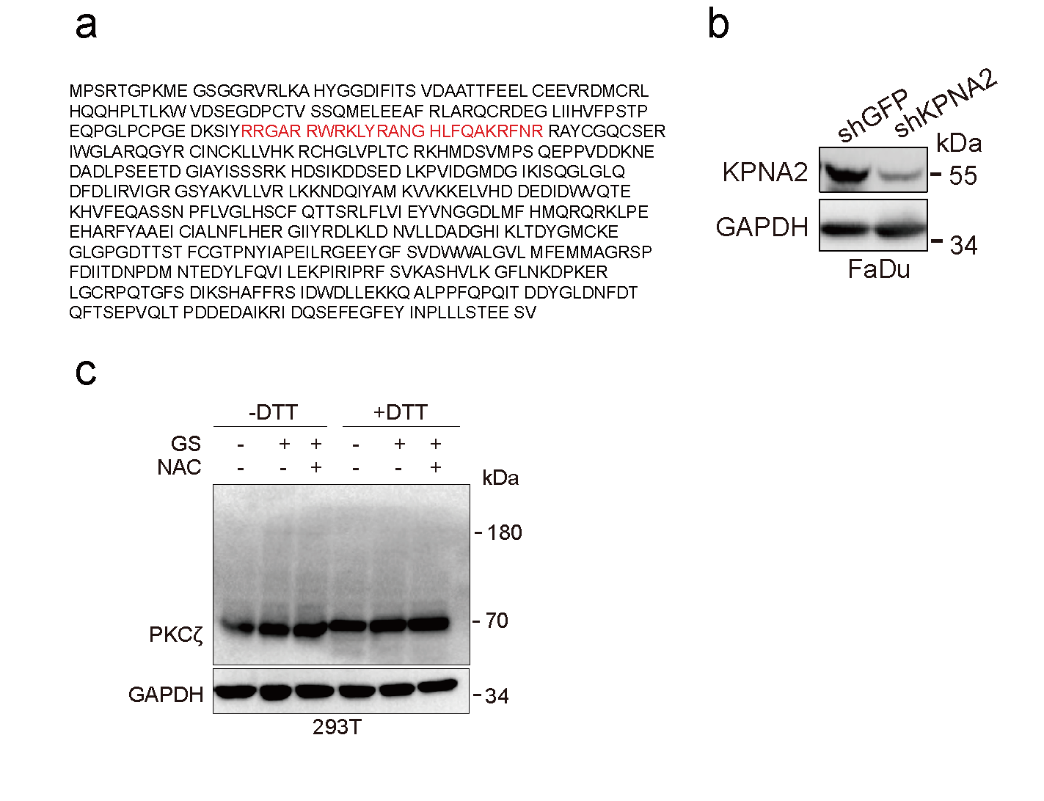


**Supplementary Figure S4** Glucose starvation could not promote PKCζ dimerization/polymerization. (a) PKCζ bears a classical nuclear localization signal sequence (NLS). The cNLS Mapper database (https://nls-mapper.iab.keio.ac.jp/cgi-bin/NLS_Mapper_help.cgi) was used for this analysis. (b) FaDu cells stably expressing *shKPNA2* or shGFP were subjected to western blot analyses. (c) HEK 293T cells were treated with or without NAC (10 mmol/L) for 10 h, followed by culture in GS condition in the presence or absence of NAC for an additional 2 h. Cells were subjected to SDS-PAGE with or without DTT followed by western blot analyses.

**
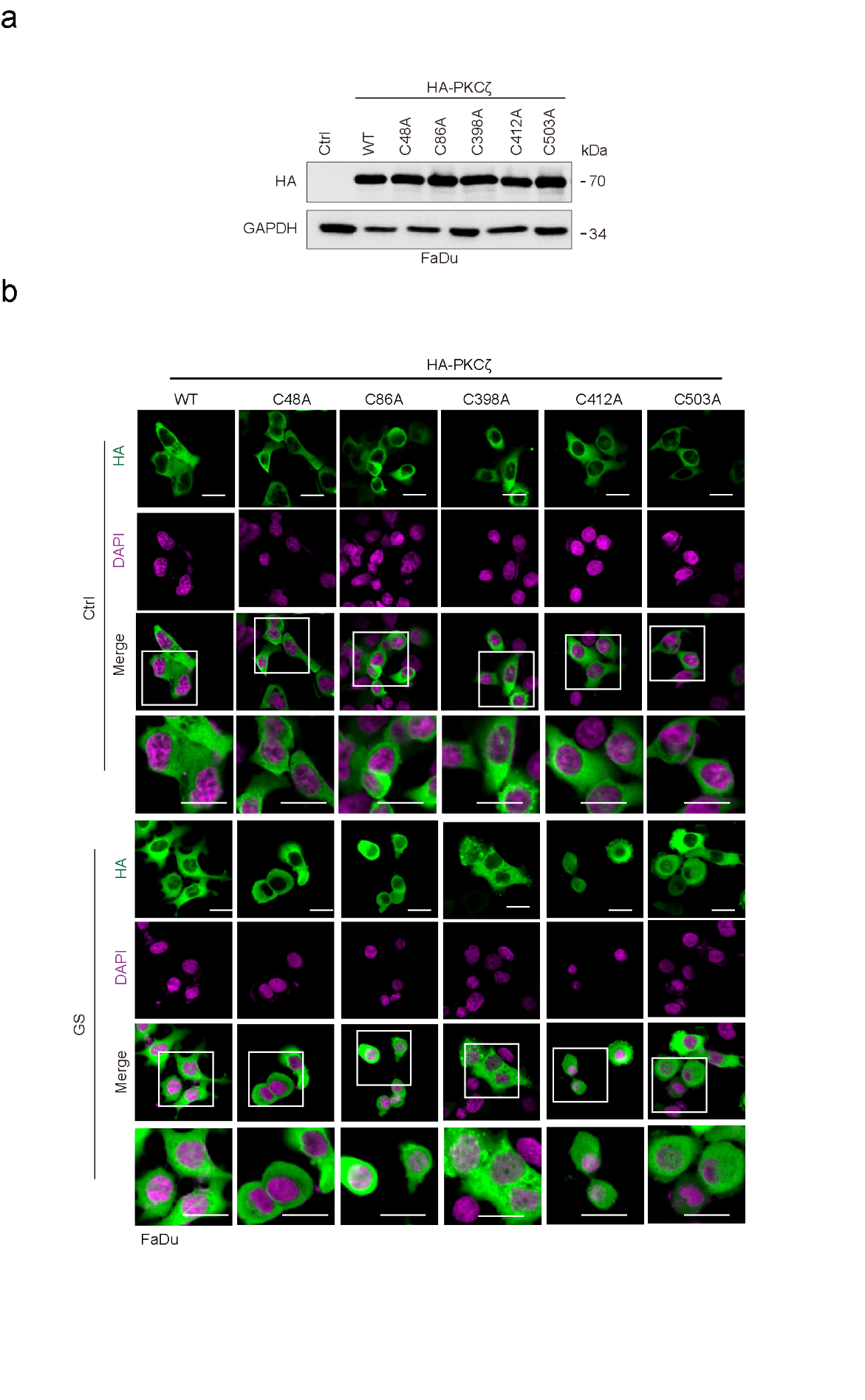
**

**Supplementary Figure S5** Cys48 is critical for PKCζ nuclear translocation upon energy stress. FaDu cells transiently expressing HA-PKCζ-WT, HA-PKCζ-C48A, HA-PKCζ-C86A, HA-PKCζ-C398A, HA-PKCζ-C412A, or HA-PKCζ-C503A were subjected to western blot analyses (a) or were grown in GS condition for 2h followed by immunofluorescence staining analyses (b).

**
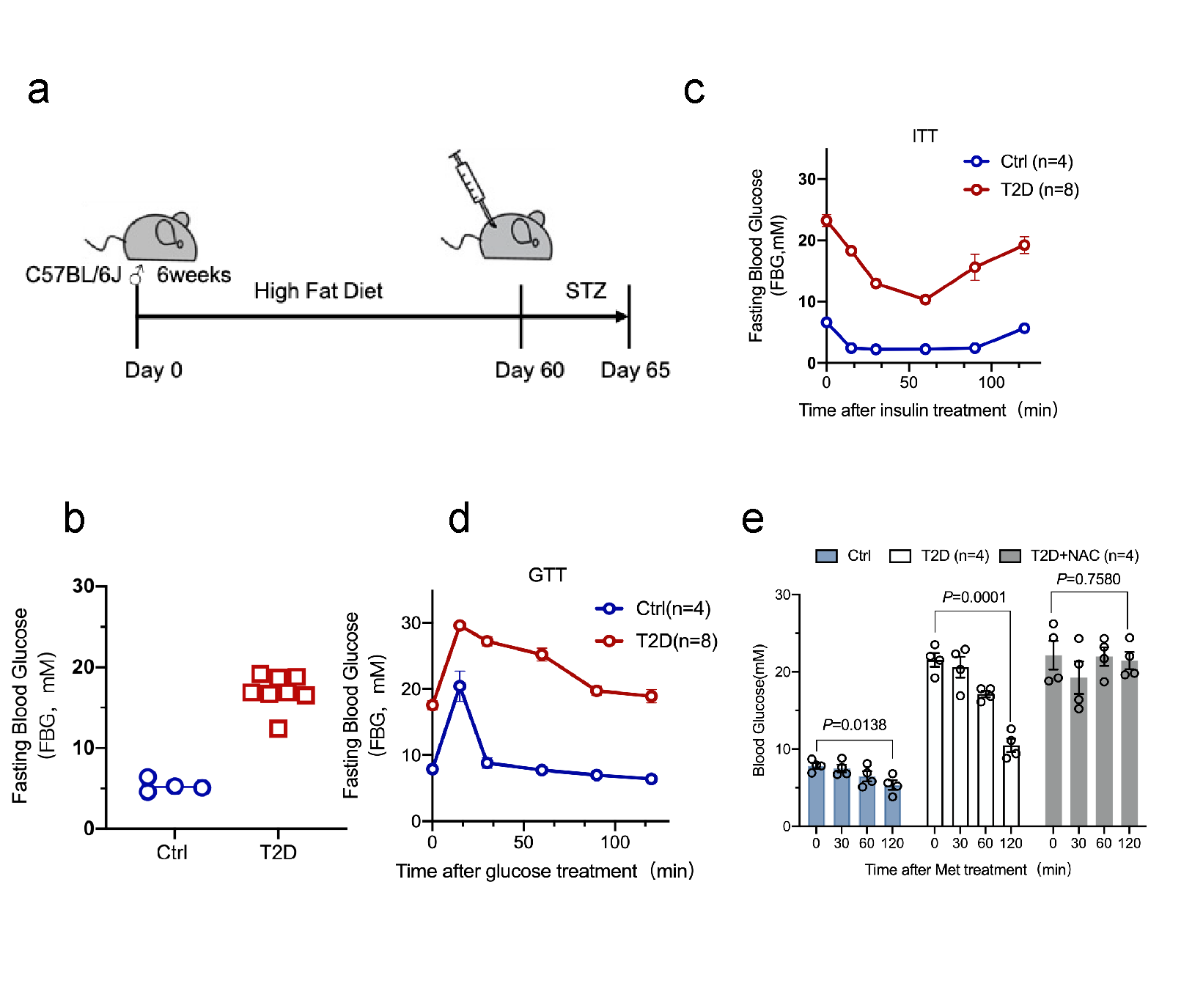
**

**Supplementary Figure S6** Diminished ROS levels impair the hypoglycemic effectiveness of Met. (a and b) Establishing the mouse model of type 2 diabetes (T2D). The 6-week-old male C57BL/6 mice were fed with a high-fat diet for 60 days, followed by intraperitoneal injection with streptozotocin (STZ, 60 mg/kg) daily (a). On day 5 after injection, mice were subjected to examining fasting blood glucose levels (b), insulin-tolerance test (ITT) (c), or glucose tolerance test (GTT) (d). (e) T2D mice were i.p. injected with NAC (200 mg/kg) daily. On day 7 after injection, mice were i.p. injected with Met (200 mg/kg) for indicated time points. Mice were subjected to examining blood glucose levels. Data were presented as mean ± SEM (c, d, and e). Comparisons were performed with unpaired two-tailed Student’s *t*-test (c).


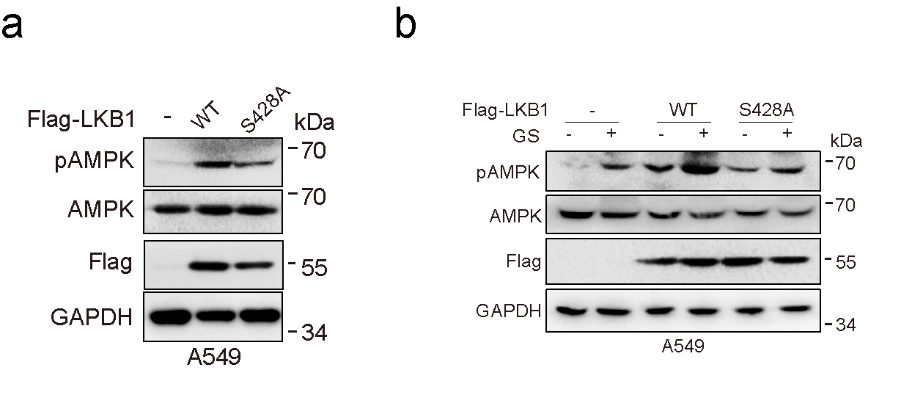


**Supplementary Figure S7** AMPK activation is more robust in A549 cells expressing wild-type LKB1 than the S428A mutant. (a) Western blot analysis of AMPK activation in A549 cells stably expressing wild-type LKB1 or the LKB1-S428A mutant under basal conditions. (b) Western blot analysis of AMPK activation in A549 cells stably expressing wild-type LKB1 or LKB1-S428A mutant grown in GS condition for 2h.
